# Canonical and Non-Canonical Roles of Connexin43 in Cardioprotection

**DOI:** 10.3390/biom10091225

**Published:** 2020-08-23

**Authors:** Olga M. Rusiecka, Jade Montgomery, Sandrine Morel, Daniela Batista-Almeida, Raf Van Campenhout, Mathieu Vinken, Henrique Girao, Brenda R. Kwak

**Affiliations:** 1 Department of Pathology and Immunology, University of Geneva, CH-1211 Geneva, Switzerland; olga.rusiecka@unige.ch (O.M.R.); jade.montgomery@unige.ch (J.M.); sandrine.morel@unige.ch (S.M.); 2 Univ Coimbra, Institute for Clinical and Biomedical Research (iCBR), Faculty of Medicine, 3000-548 Coimbra, Portugal; daniela6almeida@gmail.com (D.B.-A.); hmgirao@fmed.uc.pt (H.G.); 3 Univ Coimbra, Center for Innovative Biomedicine and Biotechnology (CIBB), 3000-548 Coimbra, Portugal; 4 Clinical Academic Centre of Coimbra (CACC), 3000-548 Coimbra, Portugal; 5 Department of In Vitro Toxicology and Dermato-Cosmetology, Vrije Universiteit Brussel, 1090 Brussels, Belgium; raf.van.campenhout@vub.be (R.V.C.); mathieu.vinken@vub.be (M.V.)

**Keywords:** connexin, Cx43, gap junction, hemi-channel, cardioprotection, myocardial infarction, ischemia/reperfusion injury

## Abstract

Since the mid-20th century, ischemic heart disease has been the world’s leading cause of death. Developing effective clinical cardioprotection strategies would make a significant impact in improving both quality of life and longevity in the worldwide population. Both ex vivo and in vivo animal models of cardiac ischemia/reperfusion (I/R) injury are robustly used in research. Connexin43 (Cx43), the predominant gap junction channel-forming protein in cardiomyocytes, has emerged as a cardioprotective target. Cx43 posttranslational modifications as well as cellular distribution are altered during cardiac reperfusion injury, inducing phosphorylation states and localization detrimental to maintaining intercellular communication and cardiac conduction. Pre- (before ischemia) and post- (after ischemia but before reperfusion) conditioning can abrogate this injury process, preserving Cx43 and reducing cell death. Pre-/post-conditioning has been shown to largely rely on the presence of Cx43, including mitochondrial Cx43, which is implicated to play a major role in pre-conditioning. Posttranslational modifications of Cx43 after injury alter the protein interactome, inducing negative protein cascades and altering protein trafficking, which then causes further damage post-I/R injury. Recently, several peptides based on the Cx43 sequence have been found to successfully diminish cardiac injury in pre-clinical studies.

## 1. Introduction

Cardiovascular disease (CVD) is the leading cause of mortality, accounting for approximately one third of all deaths globally [1]. Ischemic heart disease, the principal component of the global CVD burden, underlies about 15 million deaths each year. Ischemia occurs when blood flow to the myocardium is restricted, such as upon the erosion or rupture of an atherosclerotic lesion, resulting in thrombus formation and the occlusion of the coronary artery [2]. Ischemia of prolonged duration induces myocardial infarction (MI). The treatment of acute MI encompasses a number of procedures that allow the rapid return of blood flow to the ischemic zone of the myocardium in an attempt to rescue viable heart muscle. Although it is essential to re-open the occluded coronary artery as early as possible, reperfusion itself paradoxically leads to further complications related to the acceleration of cell death, diminished contractile function, and ventricular arrhythmias [3]. Depending on the severity and the duration of ischemia, as well as the presence of co-morbidities such as dyslipidemia, diabetes, or hypertension, the severity of ischemia/reperfusion (I/R) injury can vary. Myocardial cell death may lead to heart failure in the long term, whereas ventricular arrhythmias may induce sudden cardiac death within an hour of the onset of symptoms. An ideal cardioprotective treatment should limit the cell death and arrhythmias induced by I/R. Many cardioprotective strategies have been successfully examined in the preclinical setting, however, no effective therapy is currently available in clinics to protect the heart from I/R injury [4,5]. In the present paper, the multiple functions of connexin43 (Cx43) were reviewed in the context of cardioprotection. Particular attention was paid to the canonical function of Cx43, namely as a protein forming gap junction channels and hemi-channels, and to its emerging non-canonical functions in mitochondria, extracellular vesicles (EVs), and as a platform for protein interactions. Finally, we will briefly summarize current therapeutic strategies aimed at specifically altering some of the multiple functions performed by Cx43.

## 2. Pathophysiology of Cardiac I/R Injury

Acute coronary occlusion leads to cardiac ischemia with deprivation from oxygen and nutrients, which entails a series of molecular events starting from the halt of oxidative phosphorylation, resulting in the dissipation of mitochondrial membrane potential, the depletion of ATP and ending in diminished contractile function. In response to oxygen deprivation, cells switch their metabolism towards anaerobic glycolysis, which consequently leads to the accumulation of lactate in cardiomyocytes and a reduction of intracellular pH. The Na^+^/H^+^-exchanger attempts to evacuate the excess H^+^, leading to subsequent Na^+^ accumulation and increased activity of the Na^+^/Ca^2+^-pump. This process ends in Ca^2+^ overload. Importantly, the ischemic blockade of Na^+^/K^+^-ATPase function contributes to further Na^+^ overload, which accelerates the aforementioned processes [6,7,8].

The restoration of blood flow to the ischemic myocardium induces sudden biochemical and metabolic changes that may worsen the damages occurring in the heart [6,8]. Re-oxygenation permits oxidative phosphorylation, restarting the electron transport chain. However, this leads to reactive oxygen species (ROS) production, which mediates the opening of the mitochondrial permeability transition pore (mPTP). This effect is exacerbated in response to the re-establishment of a physiological pH by lactate elimination and activation of ion exchangers. The opening of this non-selective mitochondrial pore has detrimental effects, leading to the dissipation of mitochondrial membrane potential, decreased ATP production, hyper-contracture and finally the death of cardiomyocytes. As such, the mPTP has been identified as an important target for cardioprotection [8,9].

Ultimately, the production of ROS enhances the release of pro-inflammatory signaling molecules, attracting neutrophils to the site of injury several hours after the onset of reperfusion [6]. The endothelium, which has already been activated in response to ischemia, expresses adhesion molecules, which facilitate the migration of leukocytes into the cardiac tissue, where they further contribute to cardiomyocyte death. Moreover, both activated endothelial cells (ECs) and neutrophils can serve as additional sources of ROS [8]. The detrimental effects of neutrophil recruitment on the extent of cardiac reperfusion injury have been experimentally demonstrated using blocking antibodies for cytokines and adhesion molecules [10].

## 3. Methods Used to Study Cardiac I/R Injury

The pathophysiology of I/R injury is complex and consists of many factors that contribute to its outcome and severity [8,11]. Thus, the choice of a suitable model to study the disease is critical and is dictated by the scientific question that will be addressed. Both large and small animal models are used to study I/R injury and its pathology [12]. Large animal models typically include rabbits, dogs, pigs, sheep, and rarely, primates [13,14,15], while the small models comprise mice, rats, and guinea pigs [15]. The majority of I/R studies are based on mice [15] due to the advantages offered by their easy handling and maintenance, their relatively low costs, and their short reproductive cycle [12]. Importantly, they also closely mimic human physiology and are suitable for genetic modifications allowing in-depth studies of signaling pathways involved in I/R injury. However, differences in coronary vasculature remain a significant flaw in rodent models. Moreover, surgery on small hearts is challenging and requires skilled researchers. To respect the 3R principles (Replace, Reduce, Refine) of animal research, cell-based approaches, including iPSC-derived cells and organoids, are increasingly used. Although these in vitro models have been proven useful in addressing some focal aspects of I/R, they are of limited value for understanding the intricate interplay between cardiomyocytes, endothelial cells, inflammatory cells and fibroblasts in the heart. These models will not be further discussed in this review.

### 3.1. Ex Vivo Perfused Heart as a Model for I/R Injury

**Figure 1:**
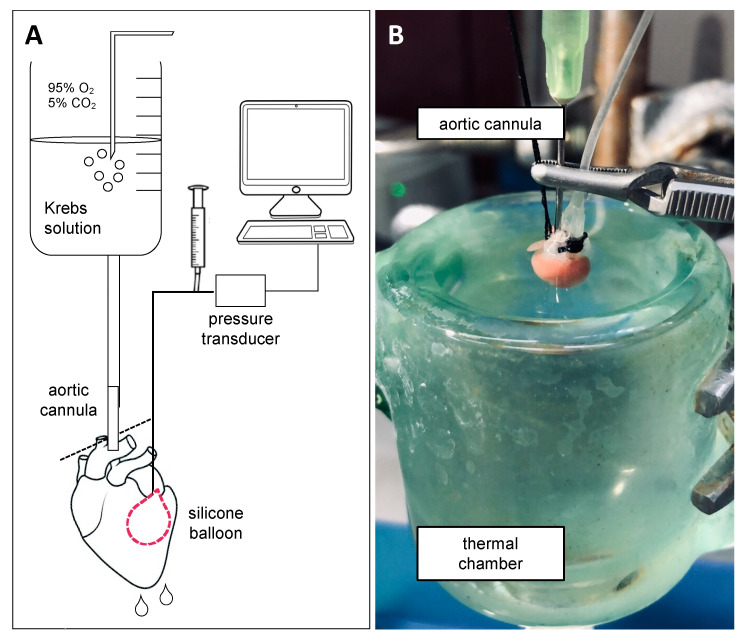
Mouse heart on a Langendorff perfusion system. (**A**) Schematic representation and (**B**) photograph of the cannulated heart. The isolated heart is cannulated via the aorta in order to be perfused with oxygenated, nutrient-rich Krebs solution. A balloon, connected to a pressure transducer, is inserted in the left ventricle for myocardial function measurements. To maintain 37 °C, the heart is immersed in a thermal chamber containing Krebs solution during the entire ex vivo procedure.

The isolated perfused heart is a reproducible and convenient model that mimics the physiological condition outside of the organism [15,16]. Ex vivo perfused hearts are most commonly studied using the Langendorff method, which allows the study of cardiac function in a viable beating heart for up to several hours [17]. This perfusion system, designed by Oskar Langendorff in 1895 [18], provides an easily accessible technique to investigate myocardial ischemia, I/R injury, preconditioning, stunning, and pharmacological interventions in the still-beating heart, together with endothelial and smooth muscle cell function [19]. The method is based on a retrograde perfusion of the heart [18,20], i.e., the heart is cannulated and perfused via the aorta, which shuts the aortic valves and directs the perfusion buffer into the coronary ostia, filling the coronary arterial vasculature. After passing the vascular bed, the perfusion buffer enters the coronary sinus in the right atrium from where it is ejected through the pulmonary artery [16,20]. As such, ventricular chambers remain non-perfused during the whole procedure [20]. The Langendorff perfusion method requires the insertion of a small silicone or latex balloon into the left ventricle, connected to a pressure transducer. The balloon is inflated with liquid to establish an end-diastolic pressure of 5–10 mmHg. Left ventricular developed pressure (LVDP) is then recorded [16,19]. Moreover, a calibrated balloon provides measurements of other cardiac function parameters, such as heart rate (HR) and pressure derivatives dP/dt_max_ and dP/dt_min_, which reflect cardiac contractility and relaxation, respectively [20]. Cardiac work is represented by the rate pressure product (RPP), which is obtained by multiplying LVDP with HR. Coronary flow is estimated by collecting the effluent within a given time period [20]. In order to maintain the physiological temperature of 37 °C, the heart is immersed in a thermostatic chamber filled with warm perfusion buffer as illustrated in Figure 1. Finally, electrocardiogram recordings performed on the perfused heart provide detailed information concerning cardiac electrical activity and conduction [21].

The vast majority of ex vivo studies on I/R injury are performed using global ischemia, which is achieved by a complete halt of perfusion [15,22]. However, it is also feasible to induce regional ischemia by placing the suture at a defined level of the coronary artery [15]. The experimental protocol of I/R injury performed on rodents consists of 15–20 min of stabilization, followed by 20–40 min of ischemia, and 60–120 min of subsequent reperfusion [16,23]. Cardiac function is monitored during each step, allowing for the assessment of the effect of ischemia on myocardial function and the functional recovery of the heart in response to I/R [24]. In order to evaluate cell death in response to ischemia, hearts that have been subjected to I/R injury are sliced and incubated with triphenyltetrazolium chloride (TTC) [25]. Due to the conversion of TTC into an insoluble red precipitate by active mitochondrial dehydrogenases, the viable myocardium is stained and can easily be distinguished from the necrotic tissue, thus allowing for the evaluation of infarct size [25,26]. These measurements require normalization to the area at risk (AAR) or to the total area of each tissue slice, depending on the protocol applied, namely regional or global ischemia, respectively [15]. Although the Langendorff model is widely used and has previously allowed for detailed studies on cardiac function before, during and after I/R, major limitations include the lack of inflammatory cells and the restricted experimental time on the ex vivo heart. An emerging ex vivo approach in the translational arena is the use of cultured living ultra-thin sections of heart tissues (myocardial slices), which maintain the myocardium’s native functions and structure and can be prepared from animal and human tissue [27].

### 3.2. In Vivo Models of Cardiac I/R Injury

**Figure 2:**
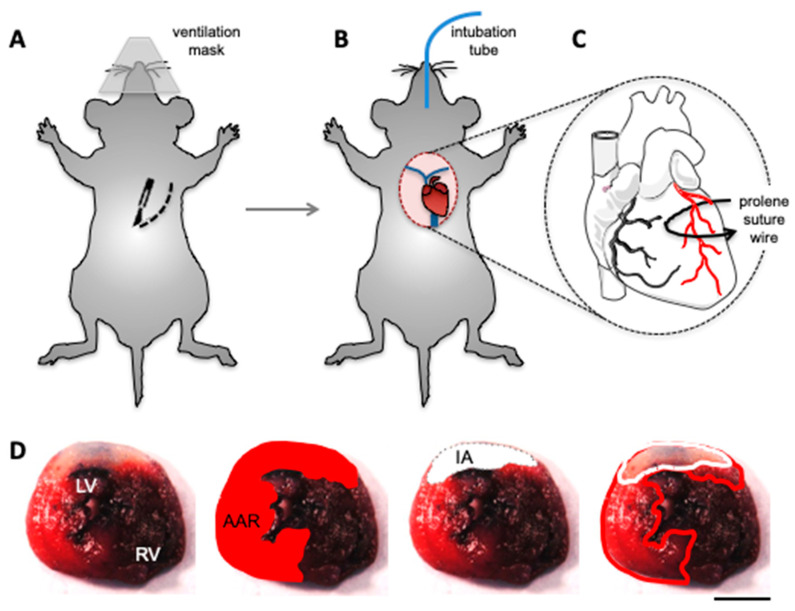
Schematic representation of the left anterior descending (LAD) ligation to induce in vivo ischemia/reperfusion (I/R) injury in mice. (**A**) After the induction of anesthesia, the mouse is placed in supine position and intubated. The chest is opened by performing a lateral incision of the left sternum side. (**B**,**C**) Separating the third and the fourth rib exposes the heart, allowing a prolene suture to be placed around the LAD in a snare that is then closed in order to induce ischemia. Reperfusion is performed 30 min later by releasing the snare. (**D**) In order to determine the area at risk (AAR), the LAD is re-occluded after reperfusion and Evans blue is injected intravenously. The dye stains all perfused tissues, including the right ventricle (RV) and part of the left ventricle (LV). The heart is sectioned into thin 1 mm slices and incubated with triphenyltetrazolium chloride (TTC) to determine the AAR (outlined in red) and the infarcted area (IA; outlined in white). Scale bar represents 50 μm.

Myocardial I/R injury is a complex injury that triggers an inflammatory response involving the activation of many cell types, including leukocytes or/and endothelial cells, which cannot be fully reproduced in ex vivo studies [8]. Thus, an in vivo approach mimicking I/R injury provides a more accurate representation of the clinical conditions found during regional myocardial ischemia with subsequent reperfusion [22]. The most popular and widely accepted approach in laboratory practice is the rodent model of I/R injury induced by transient ligation of the left anterior descending (LAD) coronary artery, commonly performed after a left-sided thoracotomy [15,22,28,29,30]. Occlusion of the LAD leads to regional myocardial ischemia [30,31]. The technique, originally designed on canine hearts, was adapted for small animals by Johns and Olson in 1954 [32]. Nowadays, (transgenic) mice are most commonly used in studies of cardiac I/R injury [33]. Figure 2A–C provides a schematic representation of the procedure performed on mice. Of note, the location of LAD ligation depends strictly on the particular infarction size aimed for in the study. In the majority of experiments, ligation is performed 1–2 mm below the left atrium resulting in 40–50% of left ventricular (LV) ischemia [33].

Arterial occlusion and infarction may be confirmed via electrocardiography based on ST-segment elevation or by observing the paling of the ischemic myocardium [12,26,33]. In most studies, the applied duration of ischemia is 20, 30, 45 or 60 min [29,33,34,35,36], after which the ligation is released and the restored blood flow to the ischemic area results in the retrieval of a pink-red colored myocardium within 20 s [33]. Depending on the study design, reperfusion is typically allowed up to 24 h [22,31,36]. Cardiac injury can then be evaluated by the examination of histopathological characteristics during the post-mortem analysis, by the measurement of markers, such as cardiac troponin I, troponin T, creatine kinase, or lactate dehydrogenase in a blood sample [22], or it can be determined from in vivo changes in ventricular function [22,29]. The latter involves the use of a Doppler ultrasound transducer, which measures the velocity of aortic blood flow [29,37]. In order to determine the AAR (Figure 2D), an intravenous injection of Evans blue is typically used [22,38]. Briefly, the LAD is re-occluded after the reperfusion period and the dye is injected into blood circulation. The dye then stains all perfused tissues, with the exception of the non-perfused area of the myocardium that defines the AAR [26,39]. The heart is then sectioned into thin 1 mm slices and incubated with TTC to determine infarct size as described above [22,25,26].

## 4. Canonical Role of Connexin43 in I/R Injury and Cardioprotection

**Figure 3:**
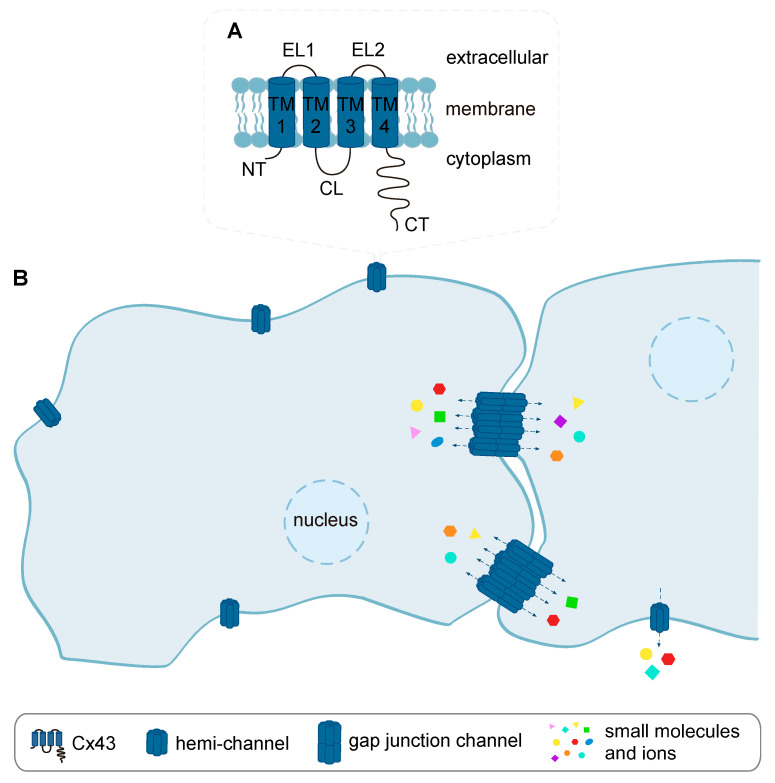
Structure of connexin (Cx) channels. (**A**) Cx topology is highly conserved, being composed of 9 structural domains, i.e., intracellular N-terminus (NT), cytoplasmic loop (CL) and C-terminus (CT), two extracellular loops (EL1 and EL2) and 4 α-helical transmembrane domains (TM1–TM4). (**B**) Gap junction channels are formed by the docking of 2 hemi-channels (or connexons) in apposed plasma membranes of adjacent cells. Each connexon is composed by the assembly of 6 connexin proteins. Gap junction channels allow the direct transfer of small molecules or ions between the cytoplasms of neighboring cells, whereas hemi-channels mediate the communication with the extracellular milieu.

Action potential propagation in the heart depends on the electrical coupling of cardiomyocytes by low resistance gap junction channels. Paradoxically, these structures also permit the cell-to-cell spread of molecules critical for cell death and survival [40]. Gap junction channels are formed by connexins (Cxs), a class of proteins that form hexameric complexes known as “connexons” or “hemi-channels” within the plasma membrane. Twenty-one different mammalian Cx genes have been reported, each coding for a transmembrane protein with similar topology whose various isoforms primarily differ in the long intracellular carboxy-terminus (CT) domain. Other Cx domains comprise four α-helical transmembrane domains (TM1–TM4), two extracellular loops (EL1 and EL2), a cytoplasmic loop (CL) between TM2 and TM3, and an intracellular amino-terminus (NT) (Figure 3A). Hemi-channels from two adjacent cells can dock together to form a gap junction channel, a functional unit that encompasses the backbone of intercellular communication (Figure 3B).

Although recent evidence suggests that the traditional direct coupling view is actually only one facet of cardiac electrical signal propagation [41,42,43], gap junctional involvement in the propagation of electrical signals in the heart remains undisputed. Of the various Cx isoforms, Cx43 is the most ubiquitously expressed and the most profoundly studied Cx due to the wide range of physiological processes in which it has been identified to play a role [40,44]. In addition to gap junctional coupling, Cx43-built channels may also act as conduits for the passage of small molecules involved in paracrine or autocrine signaling in their hemi-channel form [45]. Cx43 is subject to post-translational modifications (PTMs), which not only affect the trafficking of connexons to the cell membrane and their insertion into gap junction plaques, but also the gating properties and permeability of gap junction channels and hemi-channels [40]. Moreover, emerging data suggest channel-independent roles for the Cx family of proteins, such as the regulation of gene transcription through transcription factor hijacking or acting as a scaffolding protein for regulating the dynamics of other proteins at the cell membrane [46,47].

### 4.1. Cx43 Gap Junction- and Hemi-Channels and Cardiac I/R Injury

Cx43 is widely expressed in the healthy mammalian heart, most notably in the ventricular cardiomyocytes, but also in other cell types such as atrial cardiomyocytes, endothelial cells and fibroblasts [48]. In the last decade, compelling evidence has indicated a potential role for Cx43 in cardioprotection [49,50,51]. The role of Cx43 in cardiac I/R injury has been extensively investigated in both ex vivo [38,50,52,53,54] and in vivo models [50,55,56]. The Langendorff perfusion system has proven very useful to explore the impact of ischemic conditions on Cx43 expression, PTMs and cellular localization [52,57,58,59], while the LAD ligation model of regional I/R injury has been widely used to study the effects of pharmacological interventions involving Cx43 [54,55,56]. Many researchers have also taken advantage of transgenic mouse models [50]. As the complete genetic deletion of Cx43 was found to be lethal in mice, heterozygous Cx43^+/-^ mice have been frequently used [60]. This model, as well as a genetic replacement of Cx43 with Cx32, have provided detailed insight into the participation of Cx43 in the mechanisms leading to cardiac I/R injury [60,61].

**Figure 4:**
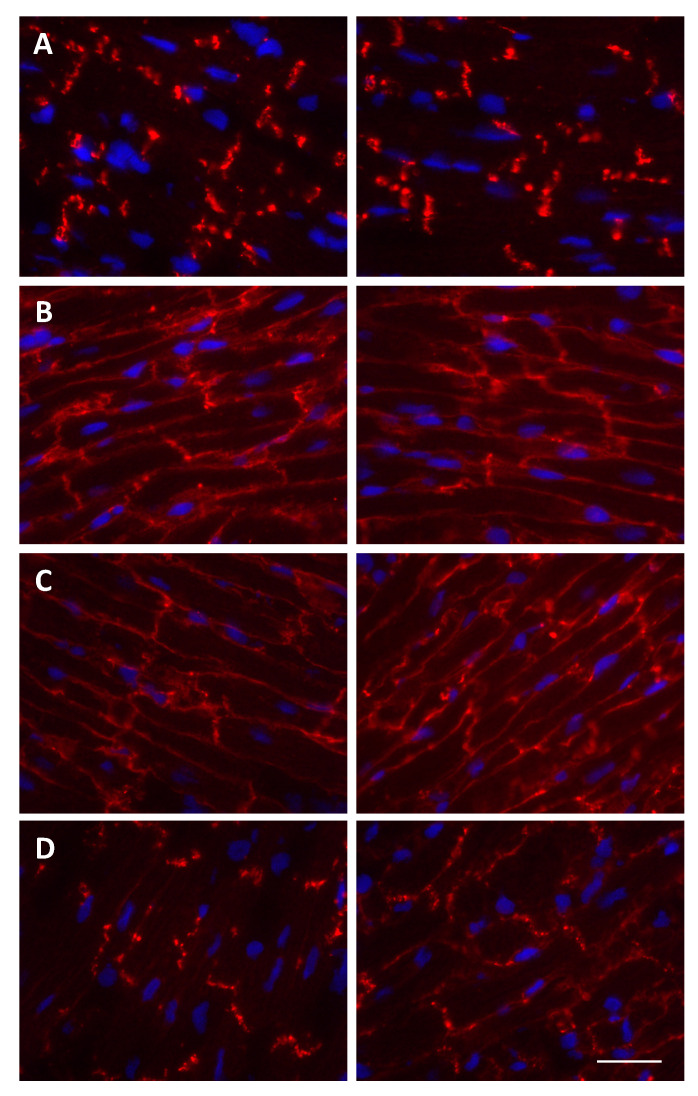
Cx43 immunofluorescent staining (in red) in Langendorff-perfused hearts subjected to ex vivo I/R. (**A**) End of stabilization period. (**B**) After 30 min of global no-flow ischemia. (**C**) After 30 min of global no-flow ischemia and 5 min reperfusion. (**D**) After 30 min of global no-flow ischemia and 60 min reperfusion. Nuclei are stained with 4′,6′-diamidino-2-phenylindole (DAPI) (in blue). Scale bar represents 20 μm.

The cellular distribution of Cx43 in a healthy ventricular cardiomyocyte is very distinct, with Cx43 heavily localized in gap junctions at polarly located intercalated disks (IDs) (Figure 4A) [48,50]. Key findings in unabrogated cardiac injury include the dephosphorylation at serine residues 325/328/330 and phosphorylation at serine 368 in the CT, as well as a redistribution of Cx43 away from the functional gap junction plaques to the lateral membrane (Figure 4B–D), complicating electrical propagation [40,48,50,57,62]. As expected, Cx43 phosphorylation and dephosphorylation during cardiac I/R is closely linked to cellular adenosine triphosphate (ATP) levels [63]. Moreover, lateralized Cx43 is mainly dephosphorylated, whereas what little is left of the phosphorylated Cx43 mostly remains at the ID [48]. This lateralization of Cx43 away from the heavily organized IDs can lead to arrhythmias, and thus further injury, in the post-ischemic heart. Beyond a simple redistribution away from its useful position, and despite the fact that the shift in phosphorylation from serine residues 325/328/330 to serine 368 reduces channel permeability [62,64], lateralized Cx43 may operate as functional hemi-channels. These hemi-channels have been implicated in causing cellular death through the release of ATP, changes in intracellular Ca^2+^ and Na^+^ concentrations, and finally cellular swelling and rupture of the plasma membrane [50,65]. The selective inhibition of Cx43 hemi-channels with the Gap19 peptide protected cardiomyocytes from volume overload, increased cell survival and reduced infarct size after cardiac I/R [66]. Frustratingly, Cx43 remaining as functional gap junctions in the ID is not without problems as well. Indeed, other research has shown that during reperfusion, signals involved in cell death and hyper-contracture can propagate through open Cx43 gap junction channels [67]. When treated with the gap junction uncoupler heptanol at the onset of reperfusion, infarct size was reduced in the ischemic pig heart [67]. Many additional studies have shown that reducing Cx43 expression or inhibiting Cx43 channels improves the outcome of cardiac I/R in vivo (reviewed in: [40]). In addition, some pharmacological interventions that are known to reduce infarct size after I/R seem to impact the phosphorylation status of Cx43. In this respect, sphingosine-1-phosphate (S1P), a constituent of high-density lipoproteins (HDL), increases Cx43 phosphorylation at serine 368, which is associated with a reduced infarct size after I/R [38]. This infarct size reduction by S1P is lost in mice in which the serine 368 is mutated to alanine, demonstrating a causal role for this phosphorylation site in cardioprotection by S1P [38]. Likewise, fibroblast growth factor 2 (FGF2) and diazoxide enhance the phosphorylation of Cx43 at serine 368 and serine 262 in isolated rat hearts [68,69], and the overexpression of mutated Cx43, with serine 262 replaced by alanine, results in augmented cell death in response to ischemia in neonatal cardiomyocytes [68]. The cardioprotective inhibition of p38 mitogen-activated protein kinase (MAPK) and pretreatment with the aCT1 peptide have also both been found to induce the phosphorylation of Cx43 at serine 368 [55,70].

Some of the earliest studies to identify the two distinct injurious events of ischemia and reperfusion in cardiac I/R injury found that reperfusion injury could be attenuated by short bursts of non-lethal ischemia and reperfusion prior to a lengthy reperfusion event [65,71,72,73,74]. Ischemic pre-conditioning and post-conditioning (IPC and PostC, respectively) of the heart made subsequent reperfusion less damaging to the tissue, and from these simple mechanical manipulations the underlying cardioprotective pathways and interactions could be explored.

IPC is known to decrease gap junctional chemical coupling during a subsequent prolonged ischemic period. Whereas IPC reduces infarct size in wild-type mice, this reduction is lost in Cx43^+/-^ counterparts [75]. Similarly, IPC-induced cardioprotection was abolished when isolated hearts from Cx43(Cre-ER(T)/fl) mice, treated with 4-hydroxytamoxifen, were submitted to global ischemia and reperfusion [76]. The latter study further demonstrated that the modification of the reperfusion injury salvage kinase (RISK) and the survivor activating factor enhancement (SAFE) signaling pathways did not contribute to the role of Cx43 in increasing tolerance to cardiac I/R injury and IPC-induced cardioprotection [76]. Interestingly, when Cx43 channels were inhibited by heptanol during IPC cycles, infarct size was not reduced, suggesting that the beneficial effects conferred by IPC rely on functional gap junctions rather than the simple presence of Cx43 [77]. Cx43 phosphorylation state analyses have furthermore revealed that IPC prevents the ischemia-induced dephosphorylation of Cx43 [78,79,80]. The IPC-induced preservation of Cx43 serines 325/328/330 phosphorylation is, at least in part, due to augmented protein kinase C (PKC) activity, since IPC-induced cardioprotection was lost and Cx43 serine 368 phosphorylation incurred at higher rates in PKC knock-out mice [81]. As further evidence towards this conclusion, IPC-induced cardioprotection was also no longer effective in mice with a targeted replacement of Cx43 by Cx32 [61].

A variety of compounds and other physical manipulations have also been found to confer cardioprotection, including diazoxide and vagus nerve stimulation [67,79,82,83,84,85]. With many of these interventions, the effects on Cx43 have been studied since Cx43 distribution is such a notable marker of post-ischemic changes in cardiomyocytes (see Figure 4).

Vagus nerve stimulation prior to reperfusion in the rat preserved Cx43 at ID localization and increased the amount of phosphorylated Cx43 present in the ventricles. In the infarcted pig heart, vagus nerve stimulation pre-conditioning and post-conditioning was able to significantly reduce infarct size and ventricular fibrillation and significantly increased the amount of Cx43 phosphorylated at serine residues 325/328/330 [62,82]. Furthermore, the administration of atropine sulfate, a muscarinic antagonist, blocked these beneficial effects, suggesting a role for acetylcholine (ACh) and the muscarinic acetylcholine receptors (mAChR) in both IPC and PostC [82,85]. The M_3_ mAChR has been shown to co-locate with Cx43 in the plasma membrane of healthy rat cardiomyocytes, and this colocalization was impaired by ischemic injury [86]. This suggests that the activated M_3_ mAChR may work directly to stabilize Cx43 in the ID during cardiac I/R injury.

Diazoxide is a potent K_ATP_ channel agonist that has been found to offer cardioprotection similar to IPC. Pharmaceutically blocking the function of K_ATP_ channels eliminated the beneficial effects of IPC in a perfused rat heart model, causing the uncoupling of gap junctions and translocation of Cx43 at similar levels to unconditioned hearts. The administration of diazoxide to unconditioned hearts meanwhile mimicked the beneficial effects of IPC [79]. Diazoxide has also been linked to increased ROS formation specifically in mitochondria [83].

The heterozygous Cx43^+/-^ mouse model, with approximately 50% of the Cx43 present compared to wild-type mice [83], has been particularly helpful to dissect the multilayered effects that Cx43 plays in reperfusion injury. Cx43^+/-^ mouse hearts and cardiomyocytes received no benefit from IPC but had the same benefits with PostC as wild-type animals [84]. Cx43^+/-^ mice also received no cardioprotection when treated with diazoxide [83,84], possibly related to the reduced treatment-induced ROS formation in Cx43^+/-^ mitochondria to less than half of that induced in wild-type mitochondria [83]. In contrast, treatment with agents that increase ROS formation outside of the mitochondria conferred cardioprotection to both wild-type and Cx43^+/-^ mouse hearts to similar degrees [83]. This suggests a primary role for mitochondria, mitochondrial Cx43, and the mitochondrial formation of ROS in IPC [50,65], but a different mechanism of action for PostC. The exact extent of the role that mitoCx43 plays in PostC, if any, is controversial and discussed further below.

### 4.2. Potential Role of Cx43 in Non-Cardiac Cells of the Heart in I/R Injury

It is increasingly recognized that cardiac I/R injury is a complex pathophysiological process with many non-cardiomyocyte populations contributing to the clinical outcome. These include the ECs of the coronary arteries and cardiac microvasculature, various types of immune cells (neutrophils, monocytes/macrophages, lymphocytes and dendritic cells), other circulating cells (platelets and possibly erythrocytes) as well as non-cardiomyocyte heart resident cells such as fibroblasts [87].

An inappropriate inflammatory response in the microcirculation may increase I/R injury. ECs are key players in the orchestration of neutrophil recruitment by controlling their rolling, adhesion, crawling and finally transmigration [88]. Extracellular ATP liberated during hypoxia or ischemia can either signal directly to purinergic receptors or, after metabolic breakdown, activate adenosine receptors. In this context, neutrophils have been shown to actively release ATP via Cx43 hemi-channels, which subsequently signal through adenosine receptors in ECs, thereby promoting endothelial barrier function and attenuating neutrophil/endothelial adhesion [89]. Furthermore, a Cx43 mimetic peptide (JM2) that targets the microtubule-binding domain of Cx43 decreased ATP release in cultured human ECs and reduced early inflammation during an in vivo foreign body response [90]. Whether the specific inhibition of Cx43 hemi-channels in ECs during cardiac I/R would exert beneficial or detrimental effects remains to be investigated.

Cardiac fibroblasts represent about 11% of total cell numbers in the adult heart and define the cardiac structure and function [91]. Their principal role is to produce extracellular matrix (ECM) proteins, including interstitial collagens and proteoglycans. In addition, they are implicated in intercellular communication by secreting soluble factors, such as cytokines and growth factors, which may contribute to phenotypic changes in cardiomyocytes in response to ischemic stress. ATP release by canine cardiomyocytes through pannexin1 channels during ischemia caused fibroblast activation and trans-differentiation into a myofibroblast phenotype [92], which may contribute to fibrotic tissue remodeling. In addition, (myo-)fibroblasts can establish direct gap junctional intercellular communication (GJIC) with cardiomyocytes [93,94], which may contribute to the arrhythmias observed after I/R. The main gap junction protein expressed in cardiac fibroblasts is Cx43. Whether changes in Cx43 expression or channel properties exert direct effects on early cardiac fibroblast phenotypic remodeling during I/R is presently unknown.

## 5. Non-Canonical Role of Connexin43 in I/R Injury and Cardioprotection

### 5.1. Mitochondrial Cx43 and Cardiac I/R Injury

Under both physiological and pathological conditions, cardiac function critically depends on energy supply, of which 90% is supplied by mitochondria [95,96]. During ischemia, the diminished oxygen level has a direct impact on mitochondria and their metabolism, making them an important target for cardioprotection [97,98]. Normally, only 4% of Cx43 in cardiomyocytes is found in mitochondria [97]. Interestingly, following two cycles of 5 min I/R IPC, a rapid increase in mitoCx43 protein levels was observed in isolated rat hearts [99]. Similar effects were noticed in mitochondria isolated from pig myocardium subjected to IPC [99]. Cardiomyocytes contain two mitochondrial subpopulations, the subsarcolemmal (SSM) and the interfibrillar (IFM) mitochondria, which have different morphology and functions [100]. SSM show a reduced oxidative phosphorylation rate compared to IFM [101] as well as increased resistance to various stress stimuli [102,103]. Moreover, SSM are more susceptible to diazoxide-induced protection against the inhibition of ATP production triggered by Ca^2+^ overload [102]. Most studies have shown that Cx43 is almost exclusively present in SSM [100,104,105]. Cx43 is encoded by the nuclear genome and the rapid increase in Cx43 in mitochondria in response to IPC is mediated by the translocase of the outer membrane (TOM) pathway [95,106]. Indeed, interaction between Cx43, TOM20 and heat shock protein 90 (Hsp90) was shown by co-immunoprecipitation in the cardiac mitochondria of both pig and rat [106]. Moreover, the treatment of isolated rat heart with geldanamycin, a drug which blocks mitochondrial import by destabilizing protein complexes with Hsp90, demonstrated the involvement of Hsp90 chaperone protein in the import of Cx43 to the SSM [106]. Once Cx43 reaches the outer mitochondrial membrane (OMM), it is further recruited to the inner mitochondrial membrane (IMM) through the translocase of the inner membrane (TIM) complex [106,107], where it locates with its CT directed towards the intermembrane space [106]. Treatment with geldanamycin also abolished the cardioprotective effects of diazoxide in porcine cardiac mitochondria, suggesting that the cardioprotection conferred by IPC is mechanistically linked to the enhanced import of Cx43 to mitochondria [106,107].

A recurring question is whether Cx43 is able to form functional channels within the mitochondrial membrane. The use of non-specific Cx43 channel blockers such as carbenoxolone, heptanol, 18-α glycyrrhetinic acid or the more specific hemi-channel blocking peptide Gap19, reduced mitochondrial K^+^ influx as well as Lucifer yellow dye uptake [100,108]. Similar effects were seen in the cardiac mitochondria isolated from mice with genetic Cx43 ablation or substitution of Cx43 with Cx32 [108]. Collectively, these observations confirm the formation of functional Cx43 hemi-channels within the mitochondrial membrane. Such hemi-channels should remain closed under physiological conditions, as their opening could dissipate the mitochondrial membrane potential and affect ATP production [100,105,106]. S-nitrosylation of SSM proteins has been described as an important post-translational modification for IPC [109]. Indeed, S-nitrosylation appears to be involved in the regulation of mitoCx43 conductance, leading to greater ROS production and K^+^ influx [109,110]. Similar to Cx43 in gap junctions, phosphorylation at serine 262 and/or serine 368 has been shown to modulate the function of mitoCx43 [80,99,100,107,111]. Although mitoCx43 is generally phosphorylated at these serine sites [107,112], it is still unknown whether Cx43 reaches the mitochondrial membrane as a phosphoprotein or whether this modification is performed via PKCε in these organelles [69].

Whether mitoCx43 plays a role in PostC is still controversial. Penna and colleagues showed that subjecting rat hearts to ischemic PostC increased the levels of anti-apoptotic Bcl-2 within the mitochondria, which was accompanied by a reduction of serine 368 phosphorylated mitoCx43 [112]. Although the reduced phosphorylation of mitoCx43 may lead to diminished ROS production, a hallmark of PostC protection [74], Schulz and colleagues demonstrated that PostC-mediated cardioprotection was preserved in Cx43^+/-^ mice [84]. However, the hypoxic PostC of the rat-derived cardiomyocyte-like cell line H9c2 led to enhanced Cx43 translocation to mitochondria, which was associated with an upregulation of Bcl-2. Moreover, the silencing of Cx43 via siRNA reduced the protective effect of hypoxic PostC through reduced ROS production [113]. However, the potential role of mitoCx43 in PostC needs further investigation.

### 5.2. Cx43 Protein Partners and Their Role in Cardiac I/R Injury-Mediated Gap Junction Remodeling

**Figure 5:**
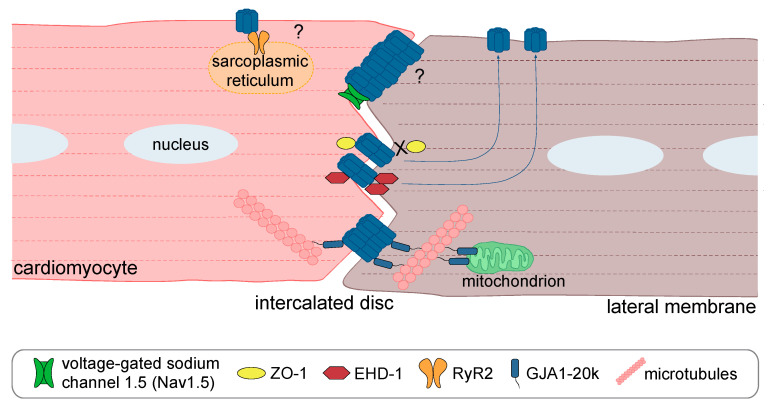
The cardiac Cx43 interactome alters in I/R-induced gap junction remodeling. A schematic representation of the subcellular localization of some relevant Cx43 interacting proteins in healthy (left; in pink) and injured (right; in brown) cardiomyocytes.

I/R-associated cardiac gap junction remodeling includes changes in Cx43 channel gating, lateralization, and degradation, largely regulated by PTMs on Cx43. Despite their multifactorial origin, most of these effects can be correlated to changes in the Cx43 interactome that are associated with pathological conditions (Figure 5). A proteomic study performed in rat hearts demonstrated that the profile of Cx43-interacting proteins alters during ischemia and I/R [114]. Importantly, the identification of a panoply of protein interactions implicated in myriad of biological processes strengthens the concept that the physiological role of Cx43 goes beyond GJIC [46]. In this light, it has been shown that Cx43 is required for the proper delivery of voltage-gated sodium channels (Nav1.5) to the cardiac ID, through a mechanism by which Cx43 captures the microtubule plus-end as part of a molecular complex [115]. Whether this non-canonical role of Cx43 can also be affected under pathological conditions, namely during I/R, remains unknown.

As expected, some of the proteins unveiled in proteomic analyses and further validated in cell-based studies are involved in protein trafficking. Accordingly, the ischemia-induced disruption of binding to zonula occludens-1 (ZO-1) allows the trafficking of Cx43 from IDs to the lateral membranes [116]. The interaction of Cx43 with 14-3-3 protein at IDs has also been associated with the ischemia-induced internalization of gap junctions [117]. Interestingly, a long non-coding RNA (lncRNA) cardiac conduction regulatory RNA (CCRR) has been demonstrated to prevent CIP85-mediated internalization and the degradation of gap junctions, which ameliorated conduction defects associated with heart diseases [118]. More recently, it was reported that the lateralization of Cx43 in cardiomyocytes subjected to ischemia is mediated by Eps15 homology domain-containing protein 1 (EHD-1) via an endocytic recycling-like mechanism. Furthermore, it has been shown that EHD-1 drives the internalization of Cx43 through a process that requires phosphorylation at serine 368, ubiquitination of Cx43 and interaction with Eps15 [119]. Another relevant cardiac Cx43-interacting protein that was demonstrated to be involved in cardiac I/R injury is the ryanodine receptor 2 (RyR2), which is vital for proper cardiac contractility. Interestingly, it was demonstrated that Cx43 hemi-channel opening relies simultaneously on calcium elevation and RyR activation [120]. On the other hand, it has been demonstrated that interaction with the 20kDa isoform (GJA1-20k), composed of a portion of TM4 together with the CT, is important to maintain the presence of full-length Cx43 at the IDs [121]. Furthermore, the cardiac-specific overexpression of GJA1-20k enhances the trafficking of Cx43 to IDs, preserving GJIC during myocardial ischemia [122].

GJIC can be regulated at the channel activity level through PTMs, albeit in a transient and reversible manner. In contrast, the degradation of Cxs constitutes a more reliable, precise and permanent mechanism of shutting down cell–cell communication. Additionally, given the unusually high turn-over of Cx43, the inhibition of its degradation rapidly results in the accumulation of Cx43, likely resulting in enhanced GJIC. Therefore, a fine-tuned regulation of Cx43′s degradation mechanisms is vital in order to ensure a well balanced communication between cardiac cells. Not surprisingly, many heart diseases are associated with the increased degradation of Cx43. In the case of ischemia or I/R, it has been demonstrated that the degradation of gap junctions in cardiomyocytes occurs mainly through autophagy [123,124]. However, the mechanisms and players in each of these clinical entities are different. After Nedd4-mediated ubiquitination, the autophagic degradation of Cx43 is mediated by AMP-activated protein kinase (AMPK), whereas during I/R the degradative process relies on Beclin 1. These results envisage the use of different therapeutic strategies aiming to stabilize Cx43 and maintain GJIC. Besides being a subject of autophagy, it has been suggested that Cx43 can also act as a repressor of autophagy by hijacking autophagy machinery at the plasma membrane [125]. Although it has not been demonstrated to date, it is therefore plausible that Cx43 remodeling associated with heart ischemia and I/R impacts autophagic activity.

Besides direct GJIC, cardiac cells can resort to EVs to exchange information [126]. According to their size, origin, and content, EVs can be divided into exosomes, microvesicles, and apoptotic bodies. Exosomes result from the fusion of multi-vesicular bodies (MVBs), formed during the endocytic pathway, with the plasma membrane and the consequent release of its intraluminal vesicles (ILVs) into the extracellular space. However, MVBs can also fuse with autophagosomes or directly with lysosomes, leading to the degradation of ILV contents. Therefore, crosstalk between exosome secretion and protein degradation has been proposed [127]. Groundbreaking studies unveiled the presence of Cx43 in EVs, in which Cx43 channels facilitate the release of vesicle content into recipient cells [128,129]. Moreover, several studies have demonstrated that the content of EVs secreted by cardiomyocytes is affected by ischemia [130,131]. Although the impact of ischemia on the sorting of Cx43 into EVs has never been addressed, it is conceivable that an interplay between EV biogenesis and autophagic degradation determines the final fate of Cx43 in ischemic cardiomyocytes.

## 6. Concluding Remarks: On the Way Towards Cx43-Targeted Strategies for Cardioprotection

Cx43 is definitely the most extensively studied isoform of this family of proteins in physiological but also pathological situations. The combination of its short half-life (1–4 h [132]) and its status as an early responder to many types of tissue injury [40,133,134] has made it an attractive target for therapeutic purposes. Depending on the pathology, tissue accessibility, and desired therapeutic effect, one may envision targeted/selective strategies to modulate different phases in the life cycle of Cx43. These include (1) targeting Cx43 mRNA levels using antisense technology resulting in the reduction of Cx43 protein levels and Cx43 hemi-channels, (2) Cx43 hemi-channel blocking by selective inhibitors, (3) size regulation of gap junction plaques by promoting hemi-channel recruitment into gap junctions, (4) size regulation of gap junction plaques by modulating Cx43 degradation mechanisms, and finally (5) GJIC increase or reduction by selective compounds [45,46]. Several Cx43-targeting agents have been tested in pre-clinical models of cardiac I/R or have reached clinical trials for various indications.

Thus, a 30-mer antisense oligodeoxynucleotide (AsODN; now named Nexagon), which knocks down Cx43 expression, has undergone Phase II clinical testing for skin wounds, in particular diabetic ulcers and non-healing venous leg ulcers [135], and has been proposed to have a beneficial effect in the context of corneal wounds. AsODN-based strategies are challenging to apply in the context of cardiac I/R, which likely explains the preference for peptide-based strategies by many researchers in pre-clinical animal models of cardiac reperfusion injury.

In 1994, Stephan Dhein reported on a new antiarrhythmic peptide (AAP10 [136]), whose derivatives (rotigaptide (ZP123) and danegaptide (ZP1609)) have since been shown in numerous cell culture and animal models to increase GJIC and cardiac conduction, and yet also reduce infarct size and cardiac I/R injury [137,138]. Furthermore, treatment with rotigaptide suppressed the ischemia-induced dephosphorylation of serine residues 297 and 368 in the Cx43 of isolated perfused rat hearts [52]. ZP1609 was also shown to reduce mitochondrial respiration and ATP production as well as to decrease the extent of cardiomyocytes suffering from hyper-contracture following I/R [139]. These cardioprotective effects, however, appeared independent of mitoCx43 [139]. Finally, a clinical proof-of-concept Phase II study on 585 patients with ST-segment elevation myocardial infarction (STEMI) showed that danegaptide, administered at least 10 min prior to reperfusion, did not improve myocardial salvage [140].

More recent strategies aimed at regulating Cx43 channels, either in gap junctions or as hemi-channels, have resorted to peptides that mimic different Cx43 protein sequences. A nonapeptide derived from the CL of Cx43, called Gap19, was shown by electrophysiology to exert a specific blocking effect on hemi-channel currents in cardiomyocytes, without blocking gap junction channels, Cx40 hemi-channels or pannexin1 channels [66]. The Gap19 peptide appeared to bind to Cx43-CT, thereby preventing intramolecular CT–CL interactions and the ischemia-induced opening of Cx43 hemi-channels. Although Gap19 protected cardiomyocytes against volume overload and cell death following I/R in vitro, in vivo experiments in mice revealed that the peptide conferred only limited cardioprotection against I/R injury [66].

aCT1 is a 25-amino acid therapeutic peptide that mimics the last nine amino acids of Cx43 (aa 374–382) preceded by a cell internalization sequence. While aCT1 was originally designed specifically to act as a competitive blocker for the Cx43–CT/ZO-1 binding interaction, resulting in large aggregate gap junction plaques when applied to cells, it has since been found to also bind sites on the Cx43–CT [70,141,142]. Moreover, pretreatment with aCT1 reduced arrhythmias after cardiac injury in mice, which was accompanied by an increase in the PKCε phosphorylation of Cx43 at serine 368. Of note, both the reduction of arrhythmias and the increase in serine 368 phosphorylation were shown to be dependent on the interaction of aCT1 with the Cx43–CT instead of the ZO-1 PDZ2 binding domain [70]. Further suggesting the antiarrhythmogenic effects of the aCT1 peptide are restricted to its Cx43-CT interactions, Kieken et al. found that elevated levels of p-cSrc, competitively driving the disassociation of Cx-43/ZO-1 complexes in the early stages of canine cardiac injury, drove Cx43 out of the IDs and into the lateral membranes, thereby increasing arrhythmogenesis [116]. Similar to the above described AsODN, aCT1 has been used in clinical trials in patients with chronic diabetic foot and venous leg ulcers where aCT1 was shown to reduce ulcer area and time-to-complete ulcer closure [133,143,144]. The compound also showed beneficial effects on scar healing of non-pathologic surgery wounds [145]. In a recent ex vivo study, the pre-ischemic infusion of aCT1 preserved LV after I/R. These cardioprotective effects of aCT1 seemed to involve a molecular mechanism independent of ZO1 binding, but likely involving the direct interaction of the peptide with the Cx43-CT [70]. Importantly, a short 9-amino acid variant of aCT1 without antennapedia sequence (called aCT11) preserved LV function when administered as post-ischemic treatment in isolated mouse hearts [70]. Although the molecular mechanism of action as well as the mode of entry to the cell remains to be elucidated in pre-clinical animal models, the aCT11 peptide may offer an attractive option for cardioprotection in patients with acute MI.

Taken together, there is compelling evidence that targeting Cx43 may be a promising cardioprotective strategy. Although the role of Cx43 is increasingly understood, much remains to be learned about the canonical and non-canonical roles of this protein in the various cell types of the heart, particularly during the course of I/R injury, in order to choose the optimal compound for cardioprotection during a defined phase of injury and disease. Similar to other cardioprotective strategies, an eventual Cx43-targeting treatment would need additional validation in clinically-relevant animal models of I/R that include the effects of aging, co-morbidities and co-medications present in MI patients [3], as several studies have shown that such conditions and medication may by themselves affect Cx43 expression and phosphorylation [40,146], and may thus lessen the desired clinical outcome.
